# Diabetisches Fußsyndrom – Teil 2: Revaskularisation, Behandlungsalternativen, Versorgungsstrukturen, Rezidivprophylaxe

**DOI:** 10.1007/s00104-020-01313-5

**Published:** 2020-11-25

**Authors:** G. Rümenapf, S. Morbach, U. Rother, C. Uhl, H. Görtz, D. Böckler, C. A. Behrendt, D. Hochlenert, G. Engels, A. Hohneck, M. Sigl, Kommission PAVK und Diabetisches Fußsyndrom der DGG e. V.

**Affiliations:** 1 Oberrheinisches Gefäßzentrum Speyer, Diakonissen-Stiftungs-Krankenhaus Speyer, Paul-Egell-Straße 33, 67346 Speyer, Deutschland; 2 Abteilung Diabetologie und Angiologie, Fachbereich , Innere Medizin, Marienkrankenhaus gGmbH Soest, Soest, Deutschland; 3 Gefäßchirurgische Abteilung, Universitätsklinikum Erlangen, Erlangen, Deutschland; 4 Klinik für Gefäßchirurgie und Endovaskuläre Chirurgie, Universitätsklinikum Heidelberg, Heidelberg, Deutschland; 5 Klinik für Gefäßchirurgie, Bonifatius Hospital Lingen, Lingen, Deutschland; 6 Klinik und Poliklinik für Gefäßmedizin, Universitätsklinikum Hamburg-Eppendorf, Hamburg, Deutschland; 7 Centrum für Diabetologie, Endoskopie und Wundheilung Köln, Köln, Deutschland; 8 Chirurgische Praxis am Bayenthalgürtel, Köln, Deutschland; 9 Abteilung für Angiologie, 1. Medizinische Klinik, Universitätsklinik Mannheim, Mannheim, Deutschland

**Keywords:** Periphere arterielle Verschlusskrankheit, Gefäßchirurgie, Fußchirurgie, Sekundärprophylaxe, Wundbehandlung, Peripheral arterial occlusive disease, Vascular surgery, Foot surgery, Secondary prophylaxis, Wound treatment

## Abstract

Das diabetische Fußsyndrom (DFS) ist die häufigste Ursache einer Majoramputation in Deutschland. Die meisten Fußläsionen werden durch repetitive Druckbelastung bei diabetischer Polyneuropathie ausgelöst. Die periphere arterielle Verschlusskrankheit (PAVK) verhindert die Wundheilung und ist Hauptrisikofaktor für Amputationen. Bei der Therapie sind die Wund- und Infektionsbehandlung sowie die zeitnahe Revaskularisation entscheidend. Der Einsatz endovaskulärer und gefäßchirurgischer Methoden ist abhängig von Verteilungsmuster und Länge der Verschlussprozesse. Beide Verfahren ergänzen sich. Die Bypasschirurgie hat beim neuroischämischen DFS einen hohen Stellenwert. Multidisziplinäre Zentren, die Revaskularisationen bei DFS anbieten, können in 90 % der Fälle eine Verbesserung der arteriellen Durchblutung erreichen und die Amputationsrate um bis zu 80 % senken. Wegen der hohen Rezidivrate diabetischer Fußläsionen sind Maßnahmen zur Sekundärprophylaxe von herausragender Bedeutung (podologische und orthopädietechnische Betreuung, Fußchirurgie).

## Abstract (en)

Diabetic foot syndrome (DFS) is the most frequent reason for major amputations in Germany. The majority of foot lesions are triggered by repetitive pressure in diabetic polyneuropathy. Peripheral arterial occlusive disease (PAOD) impairs wound healing and is the main risk factor for amputations. The treatment of wounds and infections as well as timely revascularization are decisive. The use of endovascular and vascular surgical methods depends on the distribution pattern and length of the occlusion processes. Both procedures are complementary. Bypass surgery is of great importance for neuroischemic DFS. Multidisciplinary centers that provide revascularization in DFS can achieve an improvement of arterial blood flow in 90% of the cases and reduce the amputation rate by up to 80%. Due to the high recurrence rate of diabetic foot lesions, measures for secondary prophylaxis are of exceptional importance (podological and orthopedic technical care, foot surgery).

## Lernziele

Nach der Lektüre dieses Beitrags kennen Sie …

- die Hauptgefahren und Behandlungsziele beim diabetischen Fußsyndrom (DFS),
- die Grundlagen von Wundbehandlung und Antibiotikatherapie,
- die Möglichkeiten der arteriellen Revaskularisation,
- die Alternativen zur Revaskularisation,
- die Versorgungsstrukturen und Präventionsmöglichkeiten,
- die chirurgischen Maßnahmen bei Fuß- und Zehendeformitäten beim DFS.

Im ersten Teil dieser Übersichtsarbeit [1] wurden Epidemiologie, Pathogenese, Diagnostik und Klassifikation des diabetischen Fußsyndroms (DFS) beschrieben. Im Folgenden werden Therapieziele, Grundlagen der Wund- und Infektionsbehandlung und die arteriellen Rekonstruktionsmethoden beim DFS dargestellt. Ebenso wird auf interdisziplinäre Versorgungsstrukturen und auf Präventionsmöglichkeiten eingegangen, welche die Revaskularisation langfristig erfolgreich machen. Hierzu zählen insbesondere fußchirurgische Eingriffe bei Fuß- und Zehendeformitäten.

## Hintergrund

Das DFS ist eine häufige, komplexe, kostenintensive und mitunter lebensgefährliche Komplikation des **Diabetes mellitus**

Diabetes mellitus

(DM; [2, 3]). Bei über der Hälfte aller Patienten mit DFS besteht eine relevante periphere arterielle Verschlusskrankheit (**PAVK**

**Figure 1:**
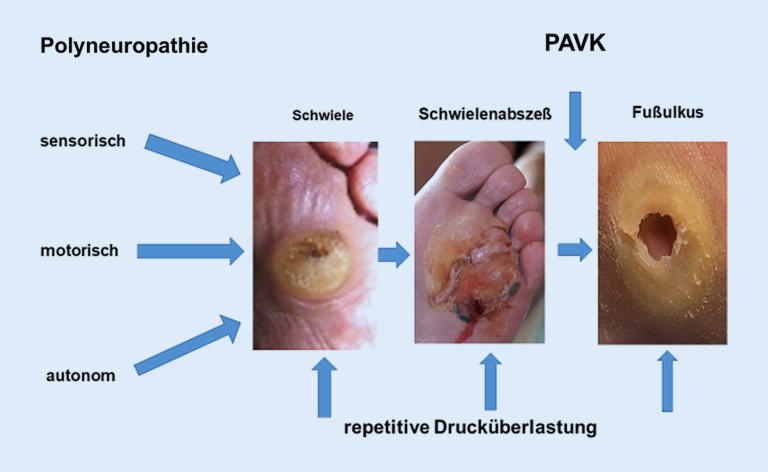

PAVK)

der Becken- und Beinarterien [4]. Zwar ist die PAVK selten die Ursache für ein Fußulkus, ihre Behandlung wird aber wichtig, wenn Fußläsionen nicht mehr heilen (Abb. 1). Leitlinienkonforme interdisziplinäre Konzepte ([5, 6, 7, 8]; **„time is tissue“**

„time is tissue“)

zur Prävention, frühzeitigen Diagnostik und rechtzeitigen Revaskularisation beim DFS haben dazu geführt, dass die Inzidenz von Majoramputationen in Deutschland kontinuierlich absinkt [9]. Die Gesamtzahl liegt aber noch immer bei weit über 10.000 Fällen pro Jahr [10]. Es ließe sich noch mehr erreichen, wenn diabetische Fußläsionen grundsätzlich ohne Zeitverlust in spezialisierte Betreuung übergeben würden, früher als bisher gefäßmedizinische Spezialisten [4] eingebunden würden und wenn noch konsequenter an eine Revaskularisation und an eine sinnvolle Rezidivprophylaxe gedacht würde.

Entscheidend für ein möglichst gutes Behandlungsergebnis ist die enge, strukturierte und vertrauensvolle Zusammenarbeit aller am Prozess beteiligten Versorgungsebenen, Berufsgruppen und medizinischen Fachdisziplinen [5, 6]. **Gefäßchirurgen**

Gefäßchirurgen

sind dabei ein wichtiger Partner. Ihre Aufgaben beim DFS sind die Revaskularisation [11] sowie im **interdisziplinären Verbund**

interdisziplinären Verbund

die Organisation der oft komplexen Wundbehandlung und Infektionsbekämpfung unter stationären Bedingungen sowie im Rahmen der Sekundärprophylaxe die Beseitigung von Fuß- und Zehendeformitäten. Die Häufigkeit von Ulkusrezidiven beträgt beim DFS zwischen 30 und 40 % innerhalb eines Jahres [3].

## Therapie

Die **Hauptgefahren**

Hauptgefahren

beim DFS sind Infektionen, Phlegmonen, Sepsis, Gangrän und Amputationen. Dies gilt auch für den diabetischen Charcot-Fuß.

Ziele einer interdisziplinären Therapie des DFS sind:

- Schmerzbekämpfung,
- Infektionskontrolle,
- arterielle Revaskularisation, Ulkusabheilung durch stadiengerechte Wundbehandlung und Druckentlastung,
- Vermeidung von Gewebeverlust und Amputationen,
- Wiederherstellung der Gehfähigkeit und Lebensqualität,
- Verhütung von Ulkusrezidiven (Sekundärprophylaxe),
- Erhalt von sozialer Integration und Selbstbestimmung,
- Vermeidung von Pflegefällen, Lebensverlängerung.

Bei Patienten mit DFS sind **leitliniengerechte Maßnahmen**

leitliniengerechte Maßnahmen

zur Senkung der Progression der PAVK und der kardiovaskulären Morbidität und Mortalität notwendig. Hierzu gehören eine **Änderung des Lebensstils**

Änderung des Lebensstils,

Rauchverbot sowie die Gabe von Hochdrucksenkern, Statinen und Thrombozytenaggregationshemmern [11]. Wichtig ist die optimale metabolische Einstellung z. B. für eine ungestörte Wundheilung oder vor einer Operation [5, 12].

## Wundbehandlung

Fußläsionen beim DFS bedürfen einer **maximalen Druckentlastung**

maximalen Druckentlastung

zum Schutz des regenerierenden Gewebes. Das gilt sowohl für neuropathische oder neuroischämische Ulzera als auch für den „offenen“ Charcot-Fuß. Falls Bettruhe nötig ist, muss auf die Druckentlastung der Fersen und Fußsohlen geachtet werden. Oft ist ein Rollstuhl unumgänglich. Beim mobilen Patienten wird die Druckentlastung durch **Gipstechniken**

Gipstechniken

(„total contact cast“, TCC), maßgefertigte oder industriell gefertigte Orthesen oder durch therapeutisches Schuhwerk erzielt [3, 5, 6, 13]. Wegen der hohen Rezidivwahrscheinlichkeit diabetischer Fußläsionen ist die **risikogerechte Schuhversorgung**

risikogerechte Schuhversorgung

von höchster Bedeutung (s. unten).

Entscheidend ist eine **stadiengerechte Wundbehandlung**

stadiengerechte Wundbehandlung

mit regelmäßiger Inspektion und Reinigung der Wunde, Nekrosenentfernung (Débridement) zur Reduktion der Keimlast und der Optimierung des Wundmilieus durch ein Temperatur- und Feuchtigkeitsmanagement. Die Wahl der Wundauflage wird durch das Wundstadium, die Exsudatmenge, Infektionszeichen, das Kosten-Nutzen-Verhältnis und die persönliche Erfahrung bestimmt.

Für die Überlegenheit der verschiedenen lokalen Wundtherapien [14] gibt es nur wenig Evidenz, z. B. für **Wundauflagen**

Wundauflagen,

die Sucrose-Octasulfat enthalten [15], oder die Applikation von **Patches**

Patches

aus autologen kombinierten Leukozyten, Thrombozyten und Fibrin [16]. Auch für antiseptische Verbände, lokal applizierte Antibiotika, Wachstumsfaktoren, enzymatische Präparate etc. fehlt die Evidenz [14].

Abszesse und Phlegmonen verlangen ein radikales **chirurgisches Débridement**

chirurgisches Débridement

einschließlich Minoramputationen [6, 8, 17]. Bei großen Substanzdefekten ist eine „Negative-pressure“-Wundbehandlung mit anschließender **plastisch-chirurgischer Defektdeckung**

plastisch-chirurgischer Defektdeckung

sinnvoll, z. B. mittels Spalthaut oder auch freien Lappentransplantaten [18].

## Antibiotika, Wundabstriche

Grundlagen der Infektionsbekämpfung bei DFS sind die Optimierung der Wundbehandlung, die Druckentlastung der Läsion, die Optimierung der arteriellen Durchblutung und die Gabe von Antibiotika.

Eine **Wundinfektion**

Wundinfektion

bei DFS wird nach der Klassifikation der International Working Group on the Diabetic Foot (IWGDF; [8, 19]) in vier Schweregrade eingeteilt (nichtinfiziert, milde, mäßige oder schwere Infektion), entsprechend auch der SINBAD („site“ [S], Ischämie [I], Neuropathie [N], bakterielle Infektion [B], „area“ [A], „depth“ [D]) und WIfI („wound“, „ischemia“, „foot infection“, WIFI) -Einteilung [1]. Das **Keimspektrum**

Keimspektrum

ist meist polymikrobiell. Am häufigsten wird *Staphylococcus aureus* gefunden, gefolgt von *E. coli*, Enterokokken, *Proteus* und *Pseudomonas aeruginosa*. Oberflächliche Keime sind pathogenetisch oftmals nicht relevant [19], weshalb immer Abstriche oder Biopsien aus der Tiefe der Fußläsion gewonnen werden sollten. Hier sind gramnegative Keime und Anaerobier häufiger. Bei jedem Patienten, ob ambulant oder stationär, muss nach Methicillin-resistentem *Staphylococcus aureus* (**MRSA**

MRSA)

gesucht werden.

Bei reizlosen Wunden und guter Granulation sind Antibiotika überflüssig. Milde Infektionen können für 1 bis 2 Wochen mit oralen Antibiotika ambulant behandelt werden. Wenn deutliche lokale (Rötung, Überwärmung, purulente Sekretion, übler Geruch, Lymphangitis) oder **systemische Infektionszeichen**

systemische Infektionszeichen

(Fieber, Leukozytose, Anstieg des C‑reaktiven Proteins) vorliegen, ist die intravenöse Gabe von Antibiotika unter stationären Bedingungen notwendig [19]. Die Auswahl des Antibiotikums geschieht zunächst empirisch nach Leitlinienempfehlung [5, 6, 7, 8, 19] und muss durch wiederholte **tiefe Abstriche**

tiefe Abstriche,

ggf. Biopsien mit entsprechenden **Antibiogrammen**

Antibiogrammen

überprüft werden. Entscheidend ist, ob das Antibiotikum rasch eine erkennbare Wirkung zeigt.

Die antibiotische Therapie sollte je nach Schwere der Infektion mit **Clindamycin**

Clindamycin,

ggf. in Kombination mit Cephalosporinen der 3. Generation (nur intravenös empfohlen), oder **Aminopenicillinen**

Aminopenicillinen

begonnen werden. Ebenfalls besteht hohe Evidenz für den Einsatz von Metronidazol (kombiniert mit anderen Antibiotika), Carbapenemen, Linezolid [19].

Orale Antibiotikagaben für mehr als 4 Wochen werden beim DFS in Zeiten des **„antibiotic stewardship“**

„antibiotic stewardship“

nur noch in Ausnahmefällen (z. B. Ostitis) empfohlen. Eher sollte dann das Behandlungsregime hinterfragt werden, denn die Probleme mit multiresistenten Bakterien (MRSA, Vancomycin-resistente Enterokokken [VRE], *Pseudomonas*) nehmen dadurch zu.

### Merke

- Die Säulen der Wundbehandlung beim DFS sind Nekrosektomie und Débridements, Infektionskontrolle, ein Temperatur- und Feuchtigkeitsmanagement, und die komplette Druckentlastung der Wunde.

### Cave!

- Langfristige Antibiotikagaben beim DFS sprechen gegen ein durchdachtes Behandlungsregime.

## Behandlung des Charcot-Fußes

Unverzichtbar ist die langfristige, **vollständige Ruhigstellung**

vollständige Ruhigstellung

des betroffenen Fußes, z. B. mittels Vollkontaktgips (TCC) oder Orthesen [5, 6, 7, 8, 13]. Die häufig übergewichtigen Patienten sind während dieser Zeit oft nur rollstuhlmobil, da die Mobilisation mit Gehstützen nur selten gelingt, weil die Patienten den geschädigten Fuß aufgrund der **Polyneuropathie**

Polyneuropathie

(PNP) nicht mehr als körpereigen empfinden und ihn deshalb nicht mehr schützen.

Regelmäßige klinische Kontrollen der **„Entzündungsaktivität“**

„Entzündungsaktivität“

(Rötung, Hauttemperatur), **Röntgenkontrollen**

Röntgenkontrollen

des Fußes zur Beurteilung der ossären Konsolidierung und die regelmäßige Überprüfung des verordneten Hilfsmittels sind notwendig. **Fußchirurgische Maßnahmen**

Fußchirurgische Maßnahmen

sind meist sehr komplex (z. B. Fixateur externe) und sollten von ausgewiesenen Spezialisten durchgeführt werden [13].

## Revaskularisation beim DFS

Die Stadieneinteilung der PAVK nach Fontaine oder Rutherford basiert auf den Kriterien Schmerz und Gewebeverlust [11]. Für das neuroischämische DFS ist sie unbrauchbar, weil das Kriterium „Schmerz“ fehlt.

Beim DFS betreffen ca. 70 % der Verschlussprozesse die **Unterschenkelarterien**

Unterschenkelarterien,

wobei die Fußarterien häufig erhalten sind. Oft sind mehrere Gefäßetagen befallen. Zur PAVK kommen beim DFS weitere Faktoren, welche die Ischämie des Fußgewebes verstärken, die Wundheilung stören und die PAVK wesentlich bedrohlicher machen als beim Nichtdiabetiker (Details hierzu wurden im ersten Teil dieser CME-Arbeit beschrieben [1]).

Bei **Knöchelverschlussdrücken**

Knöchelverschlussdrücken

von 40–50 mm Hg oder einem **transkutanen Sauerstoffpartialdruck**

transkutanen Sauerstoffpartialdruck

unter 25 mm Hg ist keine Wundheilung zu erwarten. In solchen Fällen sollte rasch revaskularisiert und mit konservativen Behandlungsversuchen keine Zeit verloren werden [20]. Bei bis zu 90 % der Patienten ist das möglich und Majoramputationen können in bis zu 80 % der Fälle vermieden werden [21, 22, 23]. Leider werden immer noch viele Majoramputationen ohne vorherige **Angiographie**

Angiographie

durchgeführt [24]. Der Gemeinsame Bundesausschuss (GBA) hat am 16.04.2020 (Pressemitteilung 17/2020) beschlossen, dass jeder Mensch mit Diabetes das Recht hat, vor einer Majoramputation eine Zweitmeinung einzuholen.

### Merke

- Die Stadieneinteilung der PAVK nach Fontaine oder Rutherford sind für das DFS unbrauchbar.
- Jeder Mensch mit Diabetes hat das Recht auf eine Zweitmeinung vor einer Majoramputation.

### Grundsätze

Arterielle Rekonstruktionen bei DFS sind keine kausale, sondern nur eine **symptomatische Behandlung**

symptomatische Behandlung.

Sie sollten ein vernünftiger Kompromiss zwischen Aufwand, Risiko und Ergebnis sein und möglichst lange funktionieren, denn 50 % der Patienten leben länger als 5 Jahre [25]. Wichtig ist, dass sich die gefäßmedizinischen Fachleute unterschiedlicher Fachgebiete, die je nach Verfügbarkeit bei der Behandlung mitwirken können, interdisziplinär verständigen. Nach gefäßchirurgischen Operationen beträgt die stationäre Wiedereinweisungsrate von DFS-Patienten innerhalb von 6 Wochen bis zu 30 %, was durch ein geeignetes **Entlassungsmanagement**

Entlassungsmanagement

auf ein Drittel reduziert werden kann [26].

Vor jeder Revaskularisation muss das gesamte **arterielle Gefäßsystem**

arterielle Gefäßsystem

des Beckens und der Beine gefäßmedizinisch untersucht werden. Zentrale Verschlussprozesse werden vor peripheren behoben, und es müssen nicht alle gleichzeitig behandelt werden. Zeitlicher und operativer Aufwand sollten möglichst gering sein. Deshalb ist es auch für die Gefäßchirurgie vernünftig, dem Prinzip **„endovascular first“**

„endovascular first“

zunächst zu folgen, bevor Bypässe angelegt werden (s. unten). Bei Mehretagen-PAVK und hohem perioperativem Risiko bieten sich **Hybrideingriffe**

Hybrideingriffe

an (s. unten).

**Aortoiliakale Verschlussprozesse**

Aortoiliakale Verschlussprozesse

werden unabhängig von ihrer Komplexität **primär endovaskulär**

primär endovaskulär

behandelt [27, 28]. Die Gefäßchirurgie kommt zum Einsatz, wenn dies nicht möglich ist oder erfolglos war. Die Ergebnisse sind wegen der niedrigeren perioperativen Morbidität schlechter als die gefäßchirurgischen (5-Jahres-Offenheitsrate 60–70 % vs. über 80 %), aber akzeptabel.

**Femoropopliteale Verschlussprozesse**

**Figure 2:**
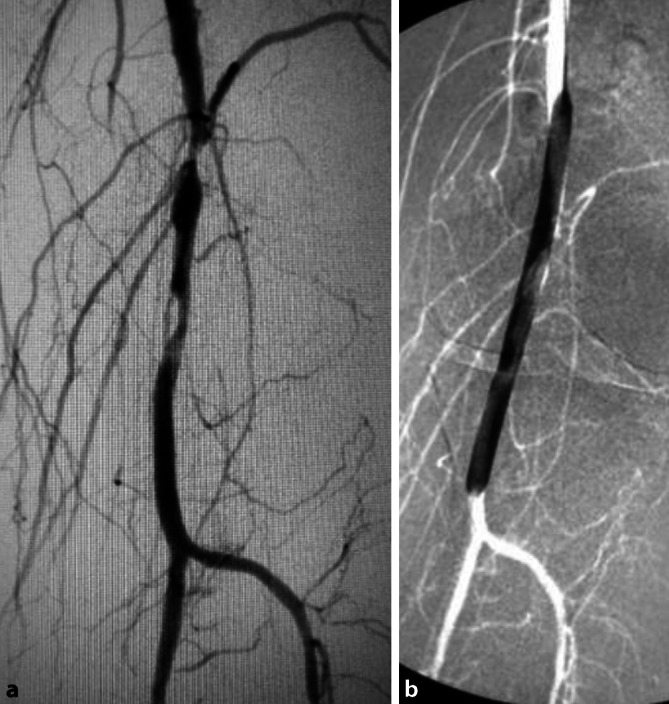

Femoropopliteale Verschlussprozesse

werden ebenfalls vorwiegend **interventionell**

interventionell

behandelt ([11, 25]; Abb. 2).

Die technische und klinische Erfolgsrate erreicht bei **kurzstreckigen femoropoplitealen Dilatationen**

kurzstreckigen femoropoplitealen Dilatationen

90 % [29], die Rekanalisationsrate über 80 %. Nach einem Jahr sind noch ca. 70 %, nach 5 Jahren 40 % der Rekonstruktionen offen [25]. **Nitinolstents**

Nitinolstents

verbessern die Ergebnisse [30].

Zeit- und Materialaufwand und die Kosten sind bei **langstreckigen Interventionen**

langstreckigen Interventionen

hoch, die mittelfristigen Ergebnisse schlecht. Dann sind femoropopliteale **Bypässe**

Bypässe

notwendig [11, 25]. Als Bypassmaterial sollte möglichst auf **körpereigene Vene**

körpereigene Vene

zurückgegriffen werden [11]. Die primäre 5‑Jahres-Offenheitsrate solcher Venenbypässe beträgt ca. 60 % oberhalb des Knies und 50 % unterhalb des Knies, der Beinerhalt ca. 80 %. Bei Verwendung von Kunststoffbypässen sind die Ergebnisse schlechter ([11, 23]; ca. 40–50 % oberhalb, 30–40 % unterhalb des Knies). Wenn keine körpereigenen Venen verfügbar sind, kann bei drohender Amputation auf **alloplastische Bypässe**

alloplastische Bypässe

trotz der wesentlich schlechteren Ergebnisse nicht verzichtet werden.

Patienten mit **„critical limb ischemia“**

„critical limb ischemia“

(CLI) in gutem Allgemeinzustand, die eine hohe Lebenserwartung und eine bypassgeeignete Vene haben, sollten bei komplexen Verschlüssen der femoropoplitealen Achse wegen der guten Langzeitoffenheit einen Bypass bekommen. Bei gefährdeten Patienten mit kurzer Lebenserwartung ist die endovaskuläre Behandlung ungefährlicher und von der Haltbarkeit her ausreichend [31].

#### Femoralisgabel

**Figure 3:**
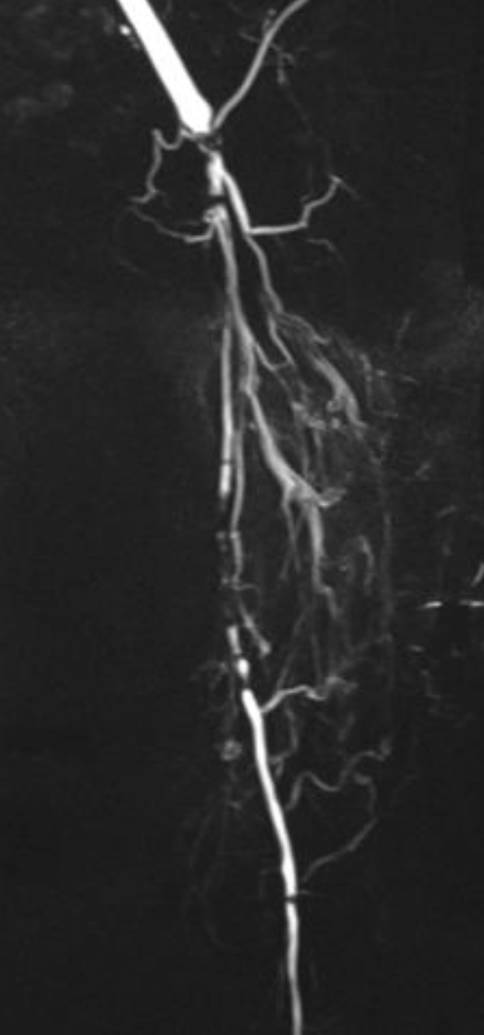

Verschlussprozesse der A. femoralis communis behindern sowohl den Einstrom in die A. femoralis superficialis als auch in die A. profunda femoris, ohne dass sich effektive Umgehungskreisläufe bilden können (Abb. 3). Auch verhindern sie endovaskuläre Maßnahmen an den Ein- und Ausstromarterien. Sie werden durch **Thrombendarteriektomie**

Thrombendarteriektomie

(TEA) mit Patcherweiterung behandelt. Aufwand und Operationszeiten sind gering, die örtliche Betäubung ist oft ausreichend. Die Beinerhaltung nach 15 Jahren beträgt über 80 % [32]. Ausschälplastiken der Leistengabel sind deshalb oft Ausgangspunkt von Hybrideingriffen (s. unten). Die **Profundaplastik**

Profundaplastik

ist zur Ausstromverbesserung bei aortoiliakalen Eingriffen notwendig. Ohne simultane Versorgung femoropoplitealer oder kruraler Verschlüsse hilft sie selten aus der CLI des Fußes heraus ([33]; Abb. 3), kann aber die Ebene einer Majoramputation nach distal verschieben.

Endovaskuläre Maßnahmen in der Femoralisgabel sind eine Alternative, wenn die Operabilität der Patienten aufgrund der Begleitkrankheiten erheblich eingeschränkt ist. Häufig wird der Profundaabgang eingeengt und Stents brechen. Stents in der Femoralisgabel behindern den bei diesen Patienten häufig notwendigen arteriellen Zugang für eine invasive Kardiodiagnostik.

### Unterschenkel- und Fußarterien

**Figure 4:**
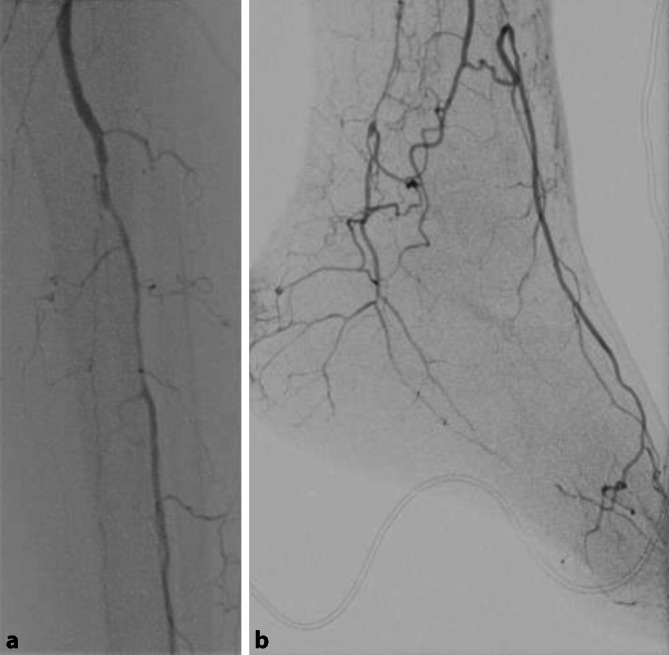

**Figure 5:**
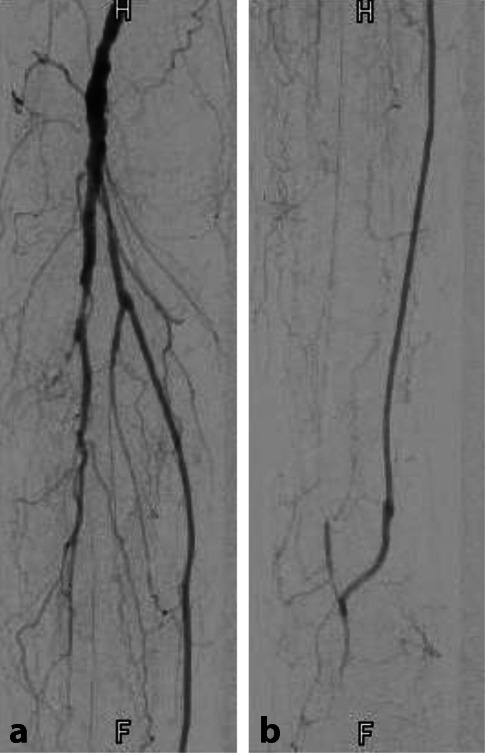

Für die Revaskularisation der Unterschenkelarterien sind offene **distale arterielle Anschlusssegmente**

distale arterielle Anschlusssegmente

notwendig. Beim DFS sind die Fußarterien häufig noch anschlussfähig (Abb. 4) und können als Zielgefäß für eine Aufdehnung oder einen pedalen Venenbypass dienen (Abb. 5).

Trotz besserer langfristiger **Offenheitsraten**

Offenheitsraten

infrapoplitealer Bypässe im Vergleich zu endovaskulären Verfahren können die **Abheilungsraten**

Abheilungsraten

der Ulzera und die **Fußerhaltungsraten**

Fußerhaltungsraten

gleich sein [34, 35]. Bei Interventionen sind das Trauma, die periprozedurale Morbidität, die Letalität sowie die Komplikationsrate geringer, dafür aber die Zahl der erforderlichen Reinterventionen erheblich höher als bei offener Gefäßchirurgie. Beim neuroischämischen DFS sollte deshalb primär endovaskulär behandelt werden [11, 28], auch wenn dies nicht „evidence based“ ist. Keines der Verfahren ist dem anderen überlegen [36], sondern sie ergänzen sich. Entscheidend sind Ausmaß und Komplexität des arteriellen Verschlussmusters, aber auch die Expertise des Behandelnden [36].

Leider gibt es für Patienten mit PAVK vom Unterschenkeltyp keine randomisierte prospektive Vergleichsstudie zwischen endovaskulärer und gefäßchirurgischer Revaskularisation. Deshalb kann die Frage nach der **besten Therapieoption**

besten Therapieoption

nicht beantwortet werden. Die nichtrandomisierten Patientenkollektive, die einer endovaskulären oder gefäßchirurgischen Behandlung zugeleitet wurden, unterscheiden sich meist vom Schweregrad der PAVK und sind daher nicht vergleichbar [37]. Auch werden immer mehr Patienten mit DFS gefäßchirurgisch behandelt, bei denen eine endovaskuläre Behandlung nicht ausgereicht hat, was die Operationen schwieriger und die Behandlungsergebnisse schlechter werden lässt.

Langstreckige Kombinationsverschlüsse der Ober- und Unterschenkelarterien werden durch **femorokrurale Bypässe**

femorokrurale Bypässe

überbrückt. Die Ergebnisse sind besser als die endovaskulärer Verfahren und entsprechen denen distalerer Bypässe [25, 38]. Langstreckige Verschlüsse der Unterschenkelarterien werden durch **„Distal-origin“-Venenbypässe**

„Distal-origin“-Venenbypässe

[39] überbrückt (z. B. popliteokrurale oder kruropedale Bypässe; Abb. 5). Ein kurzer Bypass erhöht die langfristige Offenheit der Rekonstruktion, spart Venenmaterial oder ermöglicht es, einen Bypass aus noch tauglichen Venenresten zu fertigen, wenn die Stammvenen nicht mehr komplett erhalten sind.

Die primäre **5‑Jahres-Offenheitsrate**

5‑Jahres-Offenheitsrate

kruraler und pedaler Bypässe erreicht ca. 60 %, die sekundäre bis 80 %, die Beinerhaltung liegt bei ca. 80 % [11, 25, 38] und die perioperative Letalität bei ca. 3 %. Für das Ergebnis ist es unerheblich, ob die Bypassvene entnommen und umgedreht, nach Zerstörung der Venenklappen „non-reversed“ interponiert wird oder „in situ“ verbleibt [11].

Endovaskuläre Eingriffe an den Unterschenkelarterien sind in ca. 90 % primär erfolgreich (Abb. 5), die Beinerhaltungsrate gleicht der von Bypässen [21], obwohl die primäre Offenheitsrate (ca. 50 % nach einem Jahr) wesentlich schlechter ist. Die **Restenoserate**

Restenoserate

ist mit 65 % nach 2 Jahren hoch [40]. Bei der **perkutanen transluminalen Angioplastie**

perkutanen transluminalen Angioplastie

(PTA) langstreckiger Verschlüsse der Unterschenkelarterien (ca. 20 cm) liegt die Restenoserate bei 70 % nach 3 Monaten [41].

Bisher wurden nur wenige Fallserien **infrainguinaler Revaskularisationen**

infrainguinaler Revaskularisationen

ausschließlich bei DFS publiziert. Die Ergebnisse der Bypasschirurgie sind mit oder ohne Diabetes gleich, allerdings sind die Letalität und die Gefahr der Majoramputation bei Menschen mit Diabetes erhöht [42, 43].

### Angiosomgerechte Revaskularisation

Am Fuß gibt es **definierte Durchblutungsregionen**

definierte Durchblutungsregionen

(„Angiosome“). Theoretisch sollte das Angiosom, in dem eine diabetische Fußläsion liegt, revaskularisiert werden. Gefäßchirurgisch besteht häufig keine Auswahlmöglichkeit, eventuell aber interventionell durch Rekanalisation verschlossener arterieller Anschlusssegmente. Experimentelle Studien haben das Angiosommodell für Revaskularisation beim DFS nicht bestätigt [44]. Möglicherweise verschwinden die Angiosomgrenzen, weil sich über Jahre hinweg **Kollateralsysteme**

Kollateralsysteme

zwischen den Restarterien gebildet haben.

#### Merke

- Bei arteriellen Verschlussprozessen der Beinarterien sollte zunächst eine endovaskuläre Behandlung angestrebt werden.
- Endovaskuläre und gefäßchirurgische Strategien ergänzen sich.
- Gefäßchirurgische Maßnahmen haben Priorität bei langstreckigen arteriellen Gefäßverschlüssen, und bei Verschlussprozessen der Leistengabel.

### Digitale Subtraktionsangiographie in PTA-Bereitschaft

Einer rationellen klinischen und sonographischen Diagnostik [1] folgt häufig die digitale Subtraktionsangiographie (DSA) der Unterschenkelgefäße in Therapiebereitschaft. Durch die Möglichkeit des Verfahrenswechsels hat der Gefäßchirurg das **breiteste Behandlungsspektrum**

breiteste Behandlungsspektrum

aller Gefäßmediziner. Er beschränkt sich auf die endovaskuläre Therapie unkomplizierter, prognostisch günstiger Gefäßläsionen und greift ansonsten auf gefäßchirurgische Techniken zurück. Etwa zwei Drittel aller behandlungswürdigen Läsionen der Unterschenkelarterien sind dabei endovaskulär behandelbar (Tab. 1). In ca. 25 % sind kruropedale Bypässe nötig. Nichtrekonstruierbare Verschlüsse sind selten. Simultan werden Débridements und Minoramputationen durchgeführt („Kombinationseingriffe“).

**Table 1:** 

	Patientenzahl	Prozent
Gesamt	634	100
Davon Diabetes-mellitus-Patienten	565	89
Revaskularisation nicht nötig	82	14
„Nichtrekonstruierbar“	13	2,3
PTA	320	56
Bypass	116	20
Andere offene Rekonstruktion	34	6
Débridement/Minoramputation	531	94

PTA perkutane transluminale Angioplastie

### Hybrideingriffe

Mit Hybrideingriffen werden **Mehretagenverschlüsse**

Mehretagenverschlüsse

durch eine Kombination aus offener Chirurgie und endovaskulären Maßnahmen behandelt. Am häufigsten ist die **Femoralis-TEA**

Femoralis-TEA

in Kombination mit einer Angioplastie der Ein- und Ausstromgefäße [45]. Die Ergebnisse ähneln denen der offenen Gefäßchirurgie, mit einem Beinerhalt von ca. 80 % nach 3 Jahren, bei geringerem periprozeduralem Risiko.

Alle Patienten sollten nach endovaskulärer oder gefäßchirurgischer Revaskularisation **Thrombozytenaggregationshemmer**

Thrombozytenaggregationshemmer

bekommen [11]. Vitamin-K-Antagonisten können bei komplizierten Venenbypässen nützlich sein, steigern aber die Blutungsgefahr. Der Wert einer postoperativen **Duplexkontrolle**

Duplexkontrolle

von Bypässen ist evidenzbasiert [46].

#### Merke

Bei arteriellen Verschlussprozessen der Beinarterien sollte zunächst eine endovaskuläre Behandlung angestrebt werden.

Endovaskuläre und gefäßchirurgische Strategien ergänzen sich.

Gefäßchirurgische Maßnahmen haben Priorität bei langstreckigen arteriellen Gefäßverschlüssen, und bei Verschlussprozessen der Leistengabel.

## Behandlungsalternativen bei CLI

Eine arterielle Revaskularisation ist nicht mehr möglich, wenn der Patient

**Figure 6:**
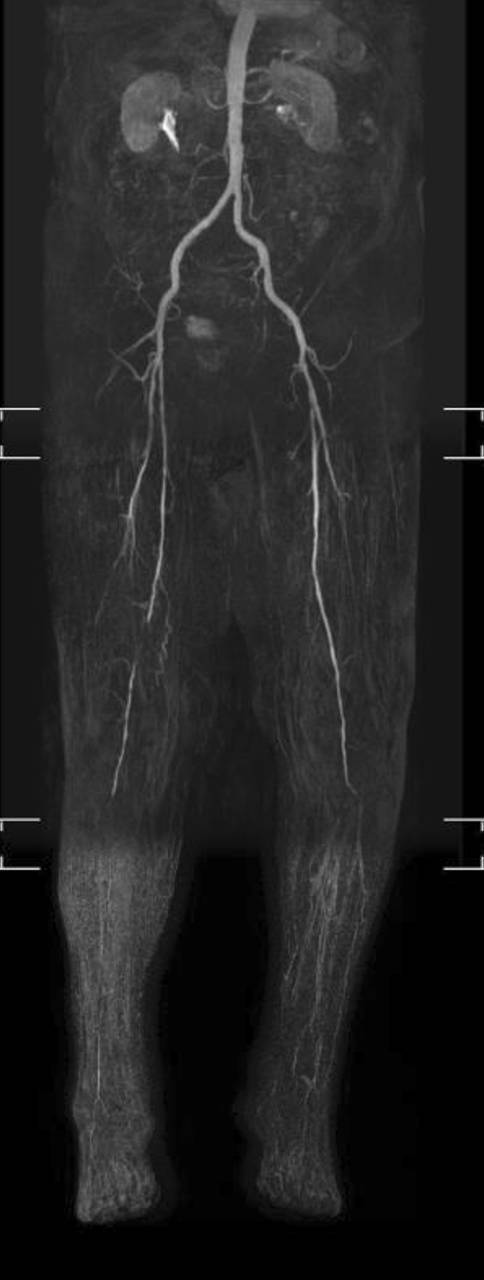

- *„zu spät“* kommt, um eine Amputation zu verhindern, wenn er
- *„zu krank“* ist, um sie zu überstehen, oder weil sie
- *technisch nicht möglich* ist („no-option CLI“; Abb. 6).

Für die Wirksamkeit von **durchblutungsfördernden Medikamenten**

durchblutungsfördernden Medikamenten

(z. B. Prostanoide), **hyperbarer Sauerstofftherapie**

hyperbarer Sauerstofftherapie

(HBOT), lumbaler Sympathikolyse (LSL), Gen- und Stammzelltherapie, endovaskulärer oder gefäßchirurgischer Arterialisation der tiefen Beinvenen etc. gibt es bei nichtrekonstruierbarer CLI nur wenig Evidenz. Sie können in Einzelfällen erfolgreich sein, werden aber von Leitlinien nicht empfohlen [11].

### Konservative Behandlung

Sie bietet sich bei Patienten mit neuroischämischem DFS an, die nicht fit für eine Revaskularisation oder Amputation sind, oder auch bei „**No-option“-Patienten**

„No-option“-Patienten,

bei denen sich die Wunden und Weichteilinfektionen durch ein geeignetes Wundmanagement „in Schach“ halten lassen.

Bisher wurde das amputationsfreie Überleben nach einem Jahr bei konservativer Therapie der CLI mit ca. 25 % beziffert [25, 47]. Neuere Publikationen (z. B. [48]) berichten aber, dass innerhalb eines Jahr bis zu 75 % der amputationsbedrohten Beine erhalten werden können. „Real-world“-Daten zeigen jedoch, dass CLI-Patienten ohne Revaskularisation ein signifikant schlechteres amputationsfreies Überleben haben [24]. Die Letalität von Menschen mit Diabetes und „no-option CLI“ ist im Vergleich zu Nichtdiabetikern signifikant erhöht, bei vergleichbarer Beinerhaltungsrate [47].

Im Rahmen der konservativen Behandlung ist die **Schmerztherapie**

Schmerztherapie

beim DFS wegen der PNP zumeist unproblematisch. Bei stark schmerzenden, therapieresistenten Wunden kann die **Majoramputation**

Majoramputation

die bessere Lösung sein [49].

#### Merke

Bei arteriell nicht rekonstruierbaren Patienten („no option“) kann die Beinerhaltungsrate durch ein geeignetes Wundmanagement 75 % nach 1 Jahr erreichen.

### Rechtzeitige Amputation

Die **primäre Majoramputation**

primäre Majoramputation

ist indiziert bei Zerstörung großer Fußanteile, bei Immobilität des Patienten, bei dementen bettlägerigen Patienten mit Kontrakturen, bei nicht beherrschbarer Infektion und bei nichttherapierbaren Schmerzen (s. oben).

Bei CLI-Patienten mit **dialysepflichtiger Niereninsuffizienz**

dialysepflichtiger Niereninsuffizienz,

schweren kardialen Begleiterkrankungen und großen Weichteilschäden raten viele Autoren wegen der schlechten Wundheilung und der hohen Mortalität zur frühzeitigen Amputation [50].

**Figure 7:**
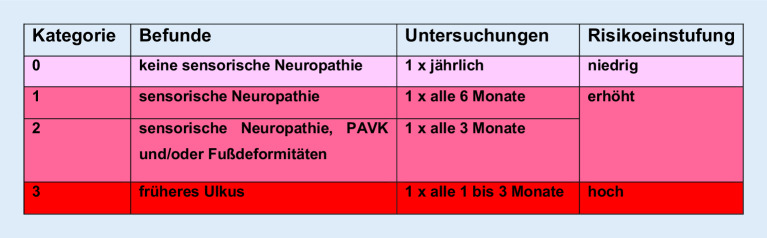

Viele DFS-Patienten mit großflächigen Läsionen der Fußsohle müssen den Fuß langfristig komplett entlasten (Abb. 7). Die Majoramputation sollte dann rechtzeitig ins Auge gefasst werden [51], um Patienten rasch, nach **zeitnaher Prothesenversorgung**

zeitnaher Prothesenversorgung,

wieder „auf die Beine zu stellen“. Je länger die Entscheidung zur Amputation herausgezögert wird, desto höher ist die perioperative Letalität und umso seltener gelingt eine Prothesenversorgung. Eine Majoramputation vermindert zwar die **Lebenserwartung**

Lebenserwartung

(5-Jahres-Mortalität 80 %) im Vergleich zu einer erfolgreichen Revaskularisation [51, 52], andererseits ist die Mortalität bei konservativer Behandlung von Patienten innerhalb eines Jahres mit mindestens 22 % auch nicht besser [53]. Eine mögliche Erklärung für die höhere Mortalität nach Amputation liegt in der höheren Krankheitsschwere und Polymorbidität der Patienten, denen dieses Vorgehen vorgeschlagen wird [53].

Nicht heilende Fußläsionen können die **Lebensqualität**

Lebensqualität

(LQ) ähnlich negativ beeinflussen wie eine Majoramputation. Eine **Unterschenkelamputation**

Unterschenkelamputation

kann sie dagegen signifikant verbessern [54]. Der Erfolg einer Rehabilitation nach Majoramputation war aus Sicht des Patienten dabei unabhängig davon, ob er eine Prothese tragen konnte oder rollstuhlmobil blieb [55]. Viele Patienten mit konservativer Therapie der CLI haben nach einem Jahr eine signifikant verbesserte LQ, obwohl sich ihr Gesundheitszustand nicht verbessert hat [56]. Die Gewöhnung an die Situation scheint also eine große Rolle zu spielen.

#### Merke

Zerstörung großer Fußanteile, Immobilität, Demenz, Kontrakturen, starke Schmerzen und nicht beherrschbare Infektionen sprechen für eine rechtzeitige Majoramputation.

## Nachbehandlung und Nachsorge

Im Rahmen einer transektoralen, **interdisziplinären Betreuung**

interdisziplinären Betreuung

von DFS-Patienten mit verteilter Versorgung und geteilter Verantwortung („shared care“) kann die Inzidenz der Majoramputationen um bis zu 80 % gesenkt werden [22]. Dabei sollten folgende Fachrichtungen vertreten und vernetzt sein:

- Hausärzte, Diabetologen,
- Gefäßmediziner (Gefäßchirurg, Angiologe, Radiologe),
- Orthopäden, Fußchirurgen, plastische Chirurgen,
- Fachpflege für Wundbehandlung,
- Orthopädieschuhmacher, Orthopädietechniker,
- Podologen,
- Schmerztherapeuten, Anästhesisten,
- Nephrologen, Kardiologen,
- Psychiater (Neglekt, Depression).

## Prävention, Rezidivprophylaxe

Der Prävention kommt eine entscheidende Bedeutung zu, um Ulzera und Amputationen zu vermeiden. Zu den Maßnahmen gehören:

- Identifikation von Risikoindikatoren für das erste Ulkusereignis,
- Identifikation von Hochrisikopatienten (vorangegangene Fußläsion oder Amputation; Befunderhebung: Suche nach Neuropathie, Pulse tasten),
- regelmäßige Untersuchung von Füßen und Schuhen (Schwielen, präulzeröse Läsionen),
- Verordnung protektiven Schuhwerks,
- Behandlung von Fuß- und Zehendeformitäten,
- regelmäßige podologische Betreuung,
- Schulung aller Beteiligten,
- psychosoziale Betreuung.

Bei Patienten mit erhöhtem Risiko (IWGDF 1 bis 3) sollten die Füße und ihre Durchblutung regelmäßig (Abb. 7) kontrolliert werden (Hausarzt, spezialisierte ambulante Einrichtung).

### Schuhversorgung

Wichtigste präventive Maßnahme ist das Tragen passender Straßen- und Hausschuhe mit **weichen Bettungen**

weichen Bettungen

zur gleichmäßigen Druckverteilung auf der Fußsohle. Schuhe und Fußbettungen müssen auf **Verschleiß**

Verschleiß

kontrolliert und häufig ersetzt werden. Druckentlastendes Material verliert rasch seine Rückstellkraft. Die **Druckmessung**

Druckmessung

im Schuh unterstützt eine verbesserte Rezidivprophylaxe von Ulzera. Eine Anleitung zur stadiengerechten Verordnung therapeutischen Schuhwerks findet sich in der Praxisleitlinie DFS der Deutschen Diabetes Gesellschaft (DDG; [5]).

Die Organisation der Fußschulung, die protektive podologische Behandlung, die stadiengerechte Schuhversorgung und die Versorgung mit Orthesen oder Prothesen sollten durch spezialisierte ambulante Zentren organisiert und überprüft werden.

### Sekundärprophylaxe

Nach Abheilen eines Fußulkus sollte beim DFS nicht von Heilung, sondern von **„Remission“**

„Remission“

gesprochen werden. [3, 5]. Trotz aller Bemühungen beträgt die Rezidivrate für Fußulzera bisher nach einem Jahr zwischen 30 und 40 % und nach 5 Jahren 65 % [3]. Mechanische Faktoren spielen eine wesentliche Rolle, z. B. **Zehendeformitäten**

Zehendeformitäten

(s. unten). Infolge wiederholter Einwirkung erhöhter Drücke und Scherkräfte während des Gehens kommt es zu Verletzungen (Abb. 1). Bereits Zehenamputationen erhöhen das Risiko für ipsi- oder kontralateraler Folgeeingriffe [13, 17] und können eine **Osteoarthropathie**

Osteoarthropathie

auslösen. Sie stören die natürliche Abrollfunktion des Fußes, verkleinern die Auflagefläche der Fußsohle und führen zu Fehlstellungen und zu einer erhöhten Druckbelastung benachbarter Strukturen (sog. **„Transferläsion“**

„Transferläsion“;

[13, 17]), was die plantaren Druckverhältnisse weiter verschlechtert. Bei proximalen Fußamputationen (Chopart, Bona-Jaeger) droht die Supinationsfehlstellung des Rückfußes mit Drucküberlastung der Fußaußenkante.

#### Cave

- Die Rezidivrate beim DFS beträgt trotz aller Bemühungen bis zu 40 % nach 1 Jahr.
- Die Revaskularisation kann beim DFS langfristig nur gute Ergebnisse bringen, wenn eine geeignete Rezidiv-Prophylaxe durchgeführt wird.

### Fußchirurgie bei Deformitäten

**Figure 8:**
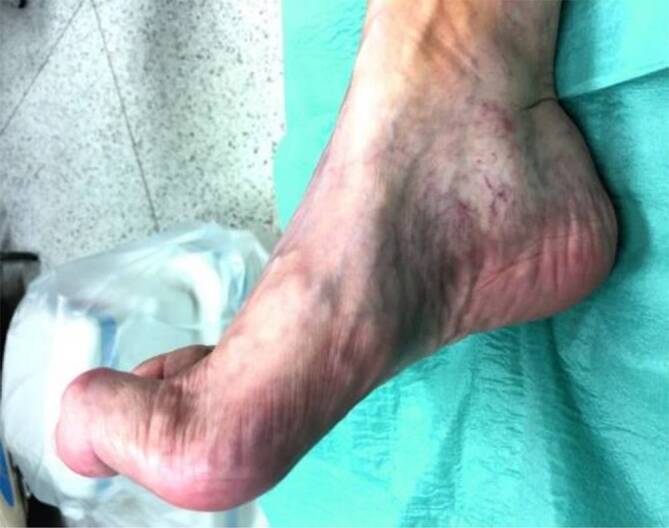

Ulkusrezidive beim DFS entstehen häufig durch **neuropathiebedingte Fehlstellungen**

neuropathiebedingte Fehlstellungen

der Füße (Abb. 8 und 9) und Zehen (Abb. 10). Sie lassen sich durch eine unkomplizierte Fußchirurgie in Form von **Release-Operationen**

Release-Operationen

an der Gastroknemiussehne („gastrocnemius release“, Abb. 11) oder den Beuge- und Strecksehnen der Zehen (Abb. 10 und 12) sehr effektiv senken [3, 13]. Minimal-invasive distale **metaphysäre Osteotomien**

metaphysäre Osteotomien

der Mittelfußköpfchen in Frästechnik bewirken eine plantare Druckentlastung. Zentren für DFS sollten diese einfachen fußchirurgischen Eingriffe anbieten.

**Figure 9:**
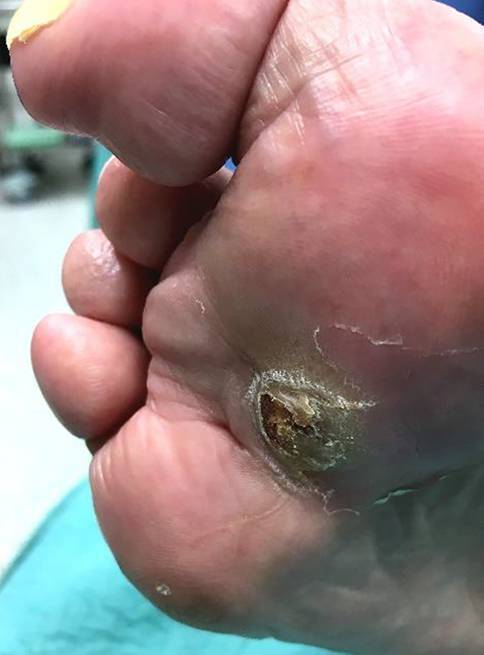

**Figure 10:**
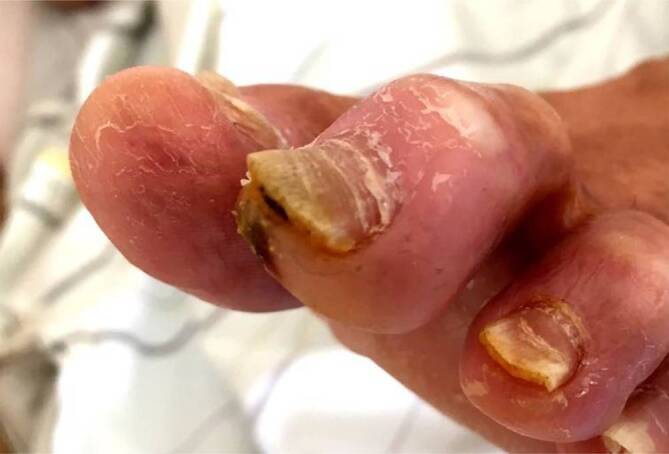

**Figure 11:**
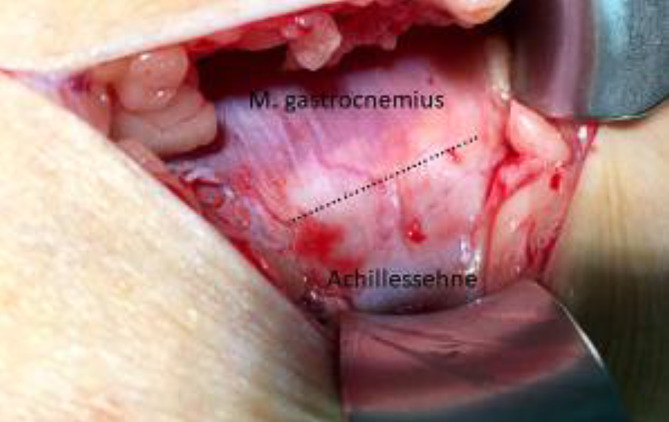

**Figure 12:**
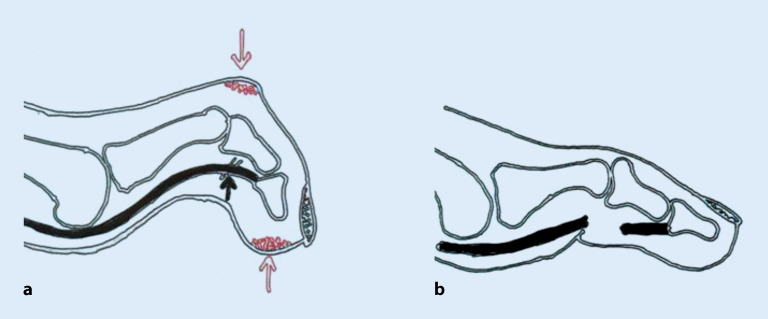

#### Merke

- Das DFS ist niemals langfristig geheilt, sondern bestenfalls in Remission.
- Fuß- und Zehendeformitäten sind häufige Ursachen für Rezidiv-Ulzera.
- Durch fußchirurgische Maßnahmen lässt sich die Rezidivrate senken.

## Fazit für die Praxis

- Beim diabetischen Fußsyndrom (DFS) muss immer an eine arterielle Durchblutungsstörung der Beine gedacht werden.
- Rechtzeitig sollte ein Gefäßspezialist zur weiteren Diagnostik und Therapie hinzugezogen werden. Durch rechtzeitige Revaskularisation können bis zu 80 % der Majoramputationen vermieden werden.
- Die Indikation zur primären Aufdehnung oder Operation hängt von der Morphologie der arteriellen Verschlussprozesse sowie der Kompetenz und der Ausstattung des behandelnden Gefäßspezialisten ab.
- Die gefäßchirurgischen Langzeitergebnisse beim DFS sind der Goldstandard, an dem sich die endovaskulären Techniken messen lassen müssen.
- Hybrideingriffe gestatten dem Gefäßchirurgen, mit einem maximalen Behandlungsspektrum das chirurgische Trauma zu vermindern und damit die perioperative Mortalität zu senken.
- Patienten mit DFS sind auch nach Abheilen der Fußläsionen zeitlebens gefährdet, ein Ulkusrezidiv zu erleiden.
- Für den langfristigen Erfolg einer Revaskularisation ist die Sekundärprophylaxe von Fußläsionen von großer Bedeutung. Hierzu zählen regelmäßige klinische Kontrolluntersuchungen, die Bereitstellung geeigneten Schuhwerks, podologische Betreuung und die operative Beseitigung von Fuß- und Zehendeformitäten.

## CME-Fragebogen

Welche Aussage zur Wundbehandlung beim diabetischen Fußsyndrom (DFS) ist richtig?

Moderne Wundauflagen ersparen die regelmäßige mechanische Wundreinigung.

Läsionen an den Füßen von Diabetikern sollten regelmäßig mechanisch belastet werden, um die Granulation der Wunden zu fördern.

Entscheidend ist eine stadiengerechte Wundbehandlung, mit regelmäßiger Inspektion und Reinigung der Wunde, Nekrosenentfernung (Débridement) zur Reduktion der Keimlast und ein Temperatur- und Feuchtigkeitsmanagement.

Für antiseptische Verbände, lokal applizierte Antibiotika, Wachstumsfaktoren, enzymatische Präparate gibt es in der Literatur höchste Evidenz.

Wundstadium, Exsudatmenge, Infektionszeichen, Kosten-Nutzen-Verhältnis und die persönliche Erfahrung spielen bei der Wahl der Wundauflage keine Rolle.

Welche Aussage zur Infektionsbekämpfung beim diabetischen Fußsyndrom (DFS) ist richtig?

Grundlage der Infektionsbekämpfung beim DFS ist die Optimierung der Wundbehandlung, mit Druckentlastung der Läsion, die Optimierung der arteriellen Durchblutung und die Gabe von Antibiotika.

Pathogenetisch relevant sind lediglich oberflächliche Keime einer Wunde. Deshalb sollte man auf Abstriche oder Biopsien aus der Tiefe der Fußläsion verzichten.

Am häufigsten wird *Pseudomonas aeruginosa* gefunden, am seltensten *Staphylococcus aureus*.

Auch wenn Infektionszeichen fehlen, muss beim diabetischen Fußulkus regelmäßig ein Antibiotikum gegeben werden.

Auch wenn deutliche lokale oder systemische Infektionszeichen bestehen, ist die orale Gabe von Antibiotika ausreichend.

Welche Aussage zur Gefäßsituation beim diabetischen Fußsyndrom (DFS) ist richtig?

Die Stadieneinteilung der peripheren arteriellen Verschlusskrankheit (PAVK) nach Fontaine oder Rutherford basieren auf den Kriterien Schmerz und Gewebeverlust und ist auch beim DFS nützlich.

Beim DFS betreffen ca. 70 % der Verschlussprozesse die Fußarterien, während die Kruralarterien häufig erhalten sind.

Bei Knöchelverschlussdrücken von 40–50 mm Hg oder einem transkutanen Sauerstoffpartialdruck unter 25 mm Hg ist keine Wundheilung zu erwarten.

Eine arterielle Revaskularisation beim DFS ist in bis zu 50 % möglich.

Majoramputationen können in bis zu 30 % der Fälle vermieden werden.

Welche Aussage zu den Prinzipien einer arteriellen Revaskularisation ist richtig?

Zentrale Verschlussprozesse werden vor peripheren behandelt.

Alle angiographisch erkennbaren arteriellen Läsionen werden gleichzeitig beseitigt.

Bei Verschlussprozessen der Unterschenkelarterien wird bei Patienten mit diabetischem Fußsyndrom (DFS) als erstes ein pedaler Bypass angelegt.

Für die Behandlung eines Patienten mit DFS ist selten eine Angiographie notwendig.

Das Recht des Patienten auf Zweitmeinung vor einer Amputation ist unsinnig, weil im Regelfall dann auch keine arterielle Rekonstruktion mehr möglich ist.

Bei vielen Patienten mit diabetischem Fußsyndrom (DFS) finden sich Verschlussprozesse der Femoralisgabel. Welche Aussage ist richtig?

Sie behindern endovaskuläre Maßnahmen über die A. femoralis communis an den Ein- und Ausstromarterien nicht.

Sie werden am besten mit einem Bypass überbrückt.

Nach erfolgter femoraler Thrombendarteriektomie (TEA) bei Verschluss der A. femoris communis beträgt die Beinerhaltung nach 15 Jahren über 80 %.

Die Profundaplastik reicht trotz simultaner Verschlüsse der A. femoralis superficialis oder der A. poplitea aus, um eine kritische Ischämie des Fußes zu beheben.

Endovaskuläre Maßnahmen sind in der Femoralisgabel ausgesprochen sinnvoll und werden gerade von Gefäßchirurgen bevorzugt.

Welche Aussage zu arteriellen Revaskularisationsverfahren beim diabetischen Fußsyndrom (DFS) trifft zu?

Hybrideingriffe haben ein höheres periprozedurales Risiko als Bypassoperationen.

Durch die Möglichkeit des intraoperativen Verfahrenswechsels oder der Kombination endovaskulärer und chirurgischer Revaskularisationsverfahren hat der Gefäßchirurg das größte Behandlungsspektrum aller Gefäßmediziner.

Endovaskuläre und offen-chirurgische Revaskularisationsverfahren ergänzen einander nicht.

Gelingt eine arterielle Revaskularisation nicht, ist eine Majoramputation die Folge.

Die konservative Behandlung von Patienten mit diabetischen Fußulzera ist indiskutabel, weil die Ergebnisse extrem schlecht sind.

Welche Aussage zum Vorgehen und Outcome beim diabetischen Fußsyndrom (DFS) trifft zu?

Beim DFS sind die Kruralarterien häufig nicht anschlussfähig und kommen als Zielgefäß für Bypässe nicht infrage.

Langstreckige Kombinationsverschlüsse der Ober- und Unterschenkelarterien werden durch langstreckige Interventionen versorgt.

Die primäre 5‑Jahres-Offenheitsrate kruraler und pedaler Bypässe erreicht ca. 60 %, die sekundäre bis 80 %, die Beinerhaltung liegt bei ca. 80 % und die perioperative Mortalität liegt bei ca. 3 %.

Bei Interventionen sind das Trauma, die periprozedurale Morbidität und Mortalität sowie die Rate von Komplikationen höher, dafür aber die Zahl der Reinterventionen geringer als nach einem Bypass.

Aktuelle Leitlinien empfehlen, beim neuroischämischen DFS primär operativ mittels Bypass zu therapieren.

Was ist bez. druckentlastender Maßnahmen zur Vermeidung eines diabetischen Ulkus korrekt?

Druckentlastende Fußbettungen in Hausschuhen haben keinen Effekt.

Fußbettungen in Straßenschuhen sind alle 2 Jahre auf Verschleiß zu kontrollieren.

Druckentlastendes Material verliert rasch seine Rückstellkraft.

Die Druckmessung im Schuh führt zu keiner besseren Ulkusrezidivprophylaxe.

Druckentlastende Bettungen im Schuhwerk sind nach erfolgreicher Revaskularisation verzichtbar.

Die primäre Majoramputation ist indiziert bei …

rasch fortschreitendem Gewebeverlust aller Zehen.

dementen Patienten.

eingeschränkter Gehfähigkeit.

starkem Ischämieschmerz bei kruraler peripherer arterieller Verschlusskrankheit (PAVK)

Immobilität mit Kniegelenkskontraktur.

Welche Aussage zu fußchirurgischen Maßnahmen am diabetischen Fuß trifft zu?

Ulkusrezidive durch neuropathiebedingte Fehlstellungen der Füße und Zehen sind beim diabetischen Fußsyndrom (DFS) sehr häufig.

Chronische plantare Ulzera über den Grundgelenken der Zehen als Folge einer Ballenfußfehlstellung sind schuhtechnisch meist beherrschbar.

Release-Operationen („gastrocnemius release“) sind wirkungslos, um Rezidivulzera beim DFS zu verhindern.

Bei Krallenzehen mit Ulzera an den Zehenkuppen hilft die minimal-invasive Durchtrennung der Strecksehnen mit einer Punktionskanüle.

Zentren für DFS sollten fußchirurgische Eingriffe lediglich in Ausnahmefällen durchführen, da die Rezidivrate an Druckläsionen der Zehen ohnehin hoch ist.

## References

[1] Rümenapf G, Morbach S, Rother U, Uhl C, Görtz H, Böckler D (2020). Kommission PAVK und Diabetisches Fußsyndrom der DGG e. V. Diabetisches Fußsyndrom – Teil 1. Definition, Pathophysiologie, Diagnostik und Klassifikation. Chirurg.

[2] Boulton AJM, Vileikyte L, Ragnarson-Tennvall G, Apequist J (2005). The global burden of diabetic foot disease. Lancet.

[3] Armstrong DG, Boulton AJM, Bus SA (2017). Diabetic foot ulcers and their recurrence. N Engl J Med.

[4] Prompers L, Schaper N, Apelqvist J (2008). Prediction of outcome in individuals with diabetic foot ulcers: focus on the differences between individuals with and without peripheral arterial disease. The EURODIALE study. Diabetologia.

[5] Morbach S, Müller E, Reike H, Risse A, Rümenapf G, Spraul M (2017). Diabetisches Fußsyndrom. Diabetol Stoffwechs.

[6] Bauer H, Germann G, Gries FA, Morbach S, Riepe G, Rothe U, Rümenapf G, Stiegler H, Tepe G, Uebel T, Weck M, Witte M (2007). Nationale VersorgungsLeitlinie Typ-2-Diabetes – Prävention und Therapie von Fußkomplikationen. Dtsch. Arztebl..

[7] Hingorani A (2016). The management of diabetic foot: a clinical practice guideline by the society of vascular surgery in collaboration with the American podiatric medical association and the society for vascular medicine. J Vasc Surg.

[8] IWGDF (2019) 2019 IWGDF guidelines on the prevention and management of diabetic foot disease. www.iwgdfguidelines.org. Zugegriffen 05.06.2020 10.1002/dmrr.3266 32176447

[9] Claessen H, Narres M, Haastert B, Arend W, Hoffmann F, Morbach S, Rümenapf G, Kvitkina T, Friedel H, Günster C, Schubert I, Ullrich W, Westerhoff B, Wilk A, Icks A (2018). Lower-extremity amputations in people with and without diabetes in Germany, 2008–2012—an analysis of more than 30 million inhabitants. Clin Epidemiol.

[10] Kröger K, Berg C, Santosa F, Malyar N, Reinecke H (2017). Lower limb amputation in Germany. Dtsch Arztebl Int.

[11] Lawall H, Huppert P, Espinola-Klein C, Rümenapf G (2016). Clinical practice guideline—the diagnosis and treatment of peripheral arterial disease. Dtsch Arztebl Int.

[12] Fesseha BK, Abularrage CJ, Hines KF (2018). Association of hemoglobin A1c and wound healing in diabetic foot ulcers. Diabetes Care.

[13] Hochlenert D, Engels G, Morbach S (2014). Das diabetische Fußsyndrom – Über die Entität zur Therapie.

[14] Game FL, Attinger C, Hartemann A (2016). IWGDF guidance on use of interventions to enhance the healing of chronic ulcers of the foot in diabetes. Diabetes Metab Res Rev.

[15] Edmonds M, Lazaro-Martinez JL, Alfayate-Garcia JM (2018). Sucrose octasulfate dressing versus control dressing in patients with neuroischaemic diabetic foot ulcers (explorer): an international, multicentre, double-blind, randomised, controlled trial. Lancet Diabetes Endocrinol.

[16] Game F, Jeffcoate W, Tarnow L, Jacobsen JL, Whitham DJ, Harrison EF, Ellender SJ, Fitzsimmons D, Löndahl M (2018). Leucopatch II trial team. Leucopatch system for the management of hard-to-heal diabetic foot ulcers in the UK, Denmark, and Sweden: an observer-masked, randomised controlled trial. Lancet Diabetes Endocrinol.

[17] Rümenapf G, Lang W, Morbach S (2009). Minoramputationen beim diabetischen Fußsyndrom. Orthopade.

[18] Meyer A, Goller K, Horch RE (2015). Results of combined vascular reconstruction and free flap transfer for limb salvage in patients with critical limb ischemia. J Vasc Surg.

[19] Lipsky BA, Senneville E, Abbas ZG (2019). IWGDF guideline on the diagnosis and treatment of foot infection in persons with diabetes.

[20] Lepäntalo M, Apelquist J, Setacci C, Ricco JB, de Donato G, Becker F, Robert-Ebadi H, Cao P, Eckstein HH, De Rango P, Diehm N, Schmidli J, Teraa M, Moll FL, Dick F, Davies AH (2011). Chapter V: diabetic foot. Clinical practice guidelines of the European society for vascular surgery. Eur J Vasc Surg.

[21] Dorros G, Jaff MR, Dorros AM, Mathiak LM, He T (2001). Tibioperoneal (outflow lesion) angioplasty can be used as primary treatment in 235 patients with critical limb ischemia: five-year follow-up. Circulation.

[22] Holstein P, Ellitsgaard N, Olsen BB, Ellitsgaard V (2000). Decreasing incidence of major amputations in people with diabetes. Diabetologia.

[23] Bisdas T, Borowski M, Torsello G (2015). Current practice of first-line treatment strategies in patients with critical limb ischemia. J Vasc Surg.

[24] Stella J, Engelbertz C, Gebauer K, Hassu J, Meyborg M, Freisinger E, Malyar NM (2020). Outcome of patients with chronic limb-threatening ischemia with and without revascularization. Vasa.

[25] Norgren L, Hiatt WR, Dormandy JA, Nehler MR, Harris KA, Fowkes FGR (2007). Inter-society consensus for the management of peripheral arterial disease (TASC II). Eur J Vasc Endovasc Surg.

[26] Rümenapf G, Geiger S, Schneider B, Amendt K, Wilhelm N, Morbach S, Nagel N (2013). Readmissions of patients with diabetes and foot ulcers after infra-popliteal bypass surgery: attacking the problem by an integrated case management model. Vasa.

[27] Kashyap VS (2008). The management of severe aortoiliac occlusive disease: endovascular therapy rivals open reconstruction. J Vasc Surg.

[28] Aboyans V, Ricco JB, Bartelink MEL (2018). 2017 guidelines ob the diagnosis and treatment of peripheral arterial diseases, in collaboration with the European society for vascular surgery (ESVS). Eur J Vasc Endovasc Surg.

[29] Muradin G, Bosch J, Stijnen T, Hunink M (2001). Balloon dilation and stent implantation for treatment of femoropopliteal arterial disease: meta-analysis. Radiology.

[30] Schillinger M, Sabeti S, Loewe C, Dick P, Amighi J, Mlekusch W (2006). Balloon angioplasty versus implantation of nitinol stents in the superficial femoral artery. N Engl J Med.

[31] Bradbury AW, Adam DJ, Bell J (2010). Bypass versus angioplasty in severe Ischaemia oft he leg (BASIL) trial: an intention-to-treat analysis of amputation-free and overall survival in patients randomized to a bypass surgery-first or a balloon angioplasty-first revascularization strategy. J Vasc Surg.

[32] Kechagias A, Ylönen K, Biancari F (2008). Long-term outcome after isolated endarterectomy of the femoral bifurcation. World J Surg.

[33] Al-Khoury G (2009). Isolated femoral endarterectomy: impact of SFA TASC classification on recurrence of symptoms and need for additional intervention. J Vasc Surg.

[34] Romiti M (2008). Meta-analysis of infrapopliteal angioplasty for chronic critical limb ischemia. J Vasc Surg.

[35] Söderström MI (2010). Infrapopliteal percutaneous transluminal angioplasty versus bypass surgery as first-line strategies in critical leg ischemia: a propensity score analysis. Ann Surg.

[36] Hinchliffe RJ, Forsythe RO, Apelquist J (2019). IWGDF Guideline on diagnosis, prognosis and management of peripheral artery disease in patients with a foot ulcer and diabetes.

[37] Lejay A, Thaveau F, Georg Y, Bajcz C, Kretz JG, Chafké N (2012). Autonomy following revascularisation in 80-year-old patients with critical limb ischemia. Eur J Vasc Endovasc Surg.

[38] Albers M, Romiti M, Brochado-Neto FC, De Luccia N, Pereira CA (2006). Meta-analysis of popliteo-to-distal vein bypass grafts for critical ischemia. J Vasc Surg.

[39] Veith FJ, Gupta SK, Samson RH, Flores SW, Janko G, Scher LA (1981). Superficial femoral and popliteal arteries as inflow sites for distal bypasses. Surgery.

[40] Haider SN (2006). Two year outcome with preferential use of infrainguinal angioplasty for critical ischemia. J Vasc Surg.

[41] Schmidt A, Ulrich M, Winkler B (2010). Angiographic patency and clinical outcome after balloon-angioplasty for extensive infrapopliteal arterial disease. Catheter Cardiovasc Interv.

[42] Paraskevas KI, Baker DM, Pompella A, Mikhailidis DP (2008). Does diabetes mellitus play a role in restenosis and patency rates following lower extremity peripheral arterial revascularization?. Ann Vasc Surg.

[43] Wallaert JB, Nolan BW, Adams J (2012). The impact of diabetes on postoperative outcomes following lower-extremity bypass surgery. J Vasc Surg.

[44] Rother U, Krenz K, Lang W, Horch RE, Schmid A, Heinz M, Meyer A, Regus S (2017). Immediate changes of angiosome perfusion during tibial angioplasty. J Vasc Surg.

[45] Dosluoglu HH, Lall P, Cherr GS, Harris LM, Dryjski ML (2010). Role of simple and complex hybrid revascularization procedures for symptomatic lower extremity occlusive disease. J Vasc Surg.

[46] Hischke S, Rieß HC, Bublitz MK, Kriston L, Schwandeberg T, Härter M, Bertges D, Debus ES, Behrendt CA (2019). Quality indicators in peripheral arterial occlusive disease treatment:a systematic review. Eur J Vasc Endovasc Surg.

[47] Lepäntalo M, Mätzke S (1996). Outcome of unreconstructed chronic critical leg ischaemia. Eur J Vasc Endovasc Surg.

[48] Marston WA, Davies SW, Armstrong B, Farbr MA, Mendes RC, Fulton JJ (2006). Natural history of limbs with arterial insufficiency and cronic ulceration treated without revascularization. J Vasc Surg.

[49] Sprengers RW, Teraa M, Moll FL, Ardine de Witt G, van der Graaf Y, Verhaar MC (2010). Quality of life in patients with no-option critical limb ischemia underlines the need for new effective treatment. J Vasc Surg.

[50] Wölfle K, Schaal J, Rittler S, Bruijnen H, Loeprecht H (2003). Infrainguinal bypass grafting in patients with end-stage renal disease and critical limb ischaemia: is it worthwhile?. Zentralbl Chir.

[51] Mustapha JA, Katzen BT, Neville RF, Lookstein RA, Zeller T, Miller LE, Jaff MR (2018). Determinants of long-term outcomes and costs in the management of critical limb ischemia: a population-based cohort study. J Am Heart Assoc.

[52] Abu Dabrh AM, Steffen M, Undavalli C, Asi N, Wang Z, Elamin MB, Conte M, Murad MH (2015). The natural history of untreated severe or critical limb ischemia. J Vasc Surg.

[53] Hoffstad O, Mitra N, Walsh J, Margolis DJ (2015). Diabetes, lower-extremity amputation, and death. Diabetes Care.

[54] Wukich DK, Ahn J, Raspovic KM, La Fontaine J, Lavery LA (2017). Improved quality of life after transtibial amputation in patients with diabetes-related fooot complications. Int J Low Extrem Wounds.

[55] Nehler MR, Coll JR, Hiatt WR, Regensteiner JG, Schnickel GT, Klenke WA, Strecker PK, Anderson MW, Jones DN, Whitehill TA, Moskowitz S, Krupski WC (2003). Functional outcome in a contemporary series of major lower extremity amputations. J Vasc Surg.

[56] Evans KK, Attinger CE, Al-Attar A (2011). The importance of limb preservation in the diabetic population. J Diabetes Complications.

